# Case of an Incidentally Found Squamous Cell Carcinoma of the Tonsil: Are We Underestimating Its Incidence?

**DOI:** 10.7759/cureus.6383

**Published:** 2019-12-14

**Authors:** Meghana Parsi

**Affiliations:** 1 Internal Medicine, Crozer-Chester Medical Center, Upland, USA

**Keywords:** tonsil, squamous cell carcinoma, head and neck cancer

## Abstract

A 65-year-old male was incidentally found to have a human papillomavirus-16 (HPV)-associated squamous cell carcinoma of the tonsil. His only major risk factor was chronic and heavy alcohol and smoking history. Fortunately, surgery, followed by chemoradiation led to clinical remission. HPV is a common and known cause of oro- and anogenital squamous cell carcinoma (SCC). However, unlike anogenital SCC, there is no screening method available for the detection of oro-genital SCC. HPV vaccination is known to prevent a majority of these cancers. However, rates of HPV vaccination have been declining in the United States, thus causing an increase in these preventable cancers. This case report highlights the need for increased HPV vaccination rates, which can only be made through adequate physician recommendations and patient education.

## Introduction

High-risk human papillomaviruses (HPV), particularly HPV-16, are known risk factors for the development of squamous cell carcinoma (SCC) of the oropharynx. Alcohol and smoking are other independent risk factors for the development of SCC. Recent data suggests that the incidence of oral SCC is increasing. A review conducted in 2012 revealed an increase in the incidence of HPV-related SCC from 38% to 59% [1]. This case describes an incidentally found SCC of the tonsil that was discovered during a routine physical exam. The patient underwent total remission with treatment. With rising incidence of HVP-related oral cancers, the question remains how this can be controlled. Unfortunately, secondary prevention is lacking, as there are no screening methods to help prevent oral cancers. This highlights the need for increased primary prevention.

## Case presentation

**Figure 1:**
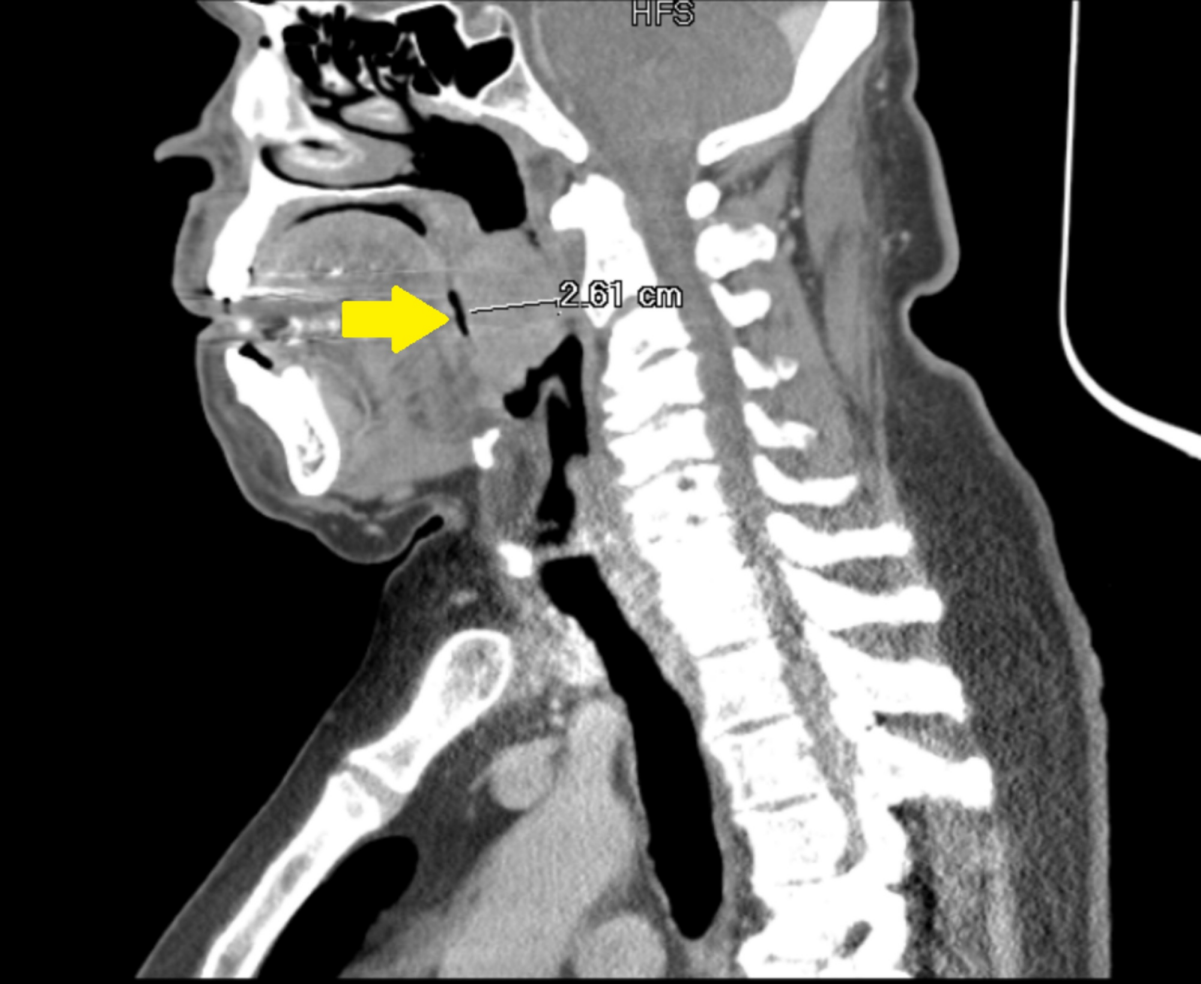
Computed tomography (CT) of the soft tissue of the neck A 2.6-cm mass can be seen obstructing the oropharyngeal airway (yellow arrow).

A 65-year-old male with a past medical history of type 2 diabetes mellitus and hypertension presented with multiple foot fractures following a fall. He endorsed to intermittent smoking history for the past 20 years along with heavy drinking, consuming more than 4-5 beers daily. Other than pain located at the site of his injury, his review of systems was negative. Complete physical exam, including head and neck exam, revealed an incidental large left tonsillar mass extending to the midline, which was friable, exophytic, and bloody. There was no palpable lymphadenopathy. The rest of his physical exam was benign, except for tenderness to the left lower extremity associated with edema and erythema. Other than pain located at the site of his foot injury, he denied fevers, weight loss, sore throat, odynophagia, dysphagia or voice changes. Lab studies were significant for a white count of 8.7 x 10*3/uL (reference range: 4.8-10.8), hemoglobin of 9.7 g/dL (13.5-17.0) and platelets of 195 x 10*3/uL (145-400). He had normal serum chemistries. Imaging of the lower extremity revealed comminuted fractures of the bases of the second, third and fourth metatarsals of the left foot. Computed tomography (CT) of the soft tissues of the neck with contrast revealed a large enhancing soft tissue mass within the left oropharynx measuring 2.6 x 2.6 x 4.7 cm in diameter. It was causing significant narrowing of oropharyngeal airway (Figure 1).

**Figure 2:**
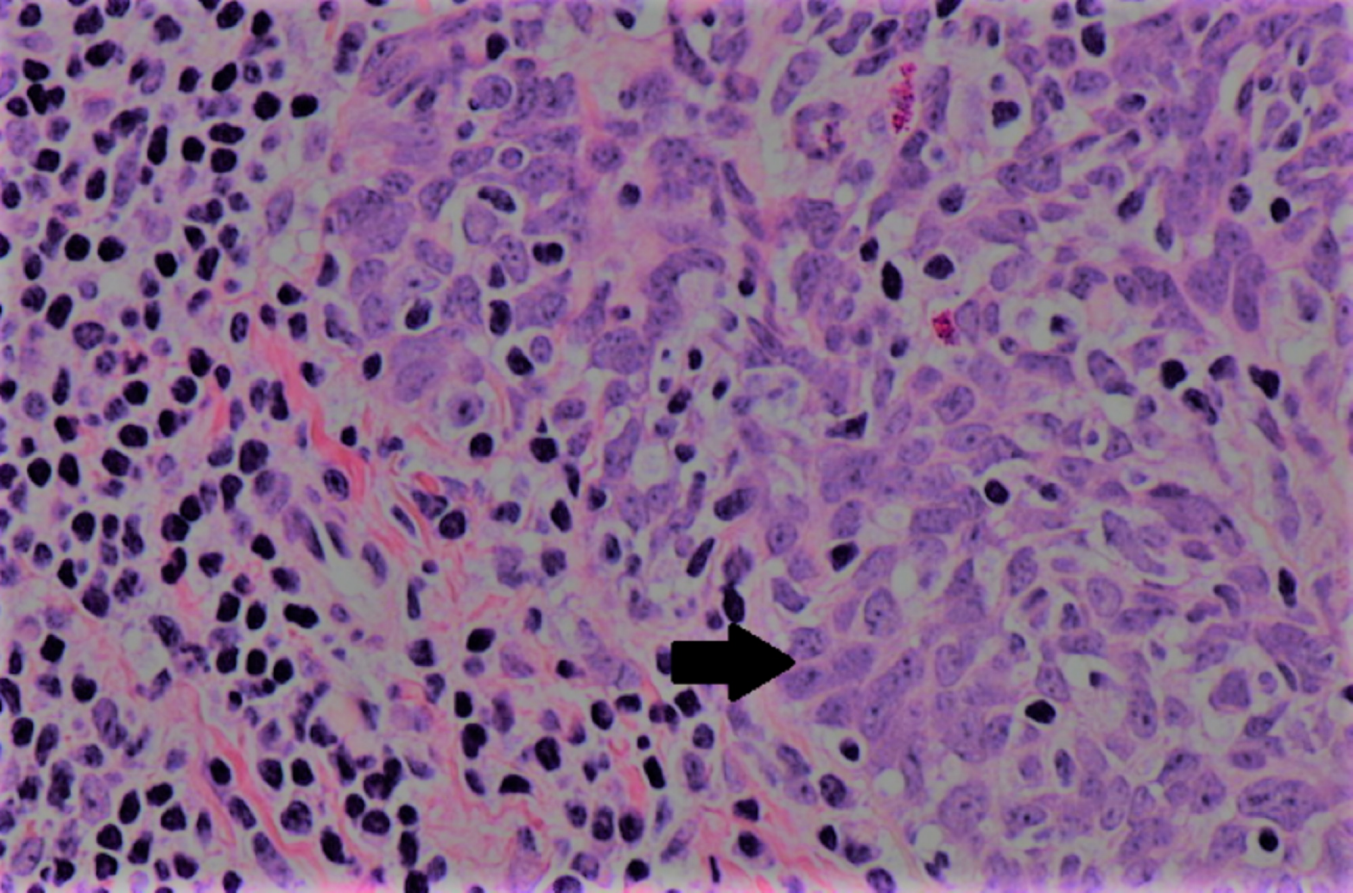
Hematoxylin and eosin (H&E) stains of non-keratinizing squamous cell carcinoma of the tonsil (NK SCC) High (400x) power images of “non-keratinizing” squamous cell histology. Tumor cells have non-prominent nucleoli and non-distinct cell borders (arrow).

A left tonsil biopsy confirmed an ulcerated noninvasive non-keratinizing squamous cell carcinoma. Immunohistochemical staining was positive for HPV-16 (Figure 2).

Further investigations revealed T3N0M0 stage disease for which he underwent a successful transoral surgical resection and elective neck dissection. There was no evidence of metastatic spread. He subsequently completed chemoradiation with six cycles of carboplatin and paclitaxel. His clinical course was complicated by ventilator-dependent respiratory failure, leading to permanent tracheostomy. Repeat scans revealed no evidence of residual disease. The patient has never been vaccinated against HPV.

## Discussion

More than 5% of all cancers in the world are caused by HPV [2]. It is responsible for all cervical cancers, a wide range of head and neck cancers and some anogenital cancers. While there are multiple high-risk HPV strains, the subtypes 16 and 18 are thought to be most carcinogenic. HPV-associated oropharyngeal cancer is thought to afflict a younger cohort of men with sexual risk factors, alcohol and tobacco use.

Screening programs utilizing the Papanicolaou smear in the United States have ensured that the incidence of cervical cancers, such as squamous cell carcinoma has drastically declined. Of note, in women aged 25 and older, the annual incidence of cervical SCC has declined to 6.5 per 100,000 women [3]. Studies now suggest that by 2020, cases of HPV-associated oropharyngeal cancers will surpass the cases of invasive cervical cancer [4].

Like in our case, incidentally found head and neck tumors allows for early identification and treatment. The current era of latest and high-tech imaging allows for the identification of incidental lesions, in patients undergoing imaging for non-oropharyngeal reasons. One study concluded that the incidence of cancer in such lesions was as high as 21% [5].

Given the high incidence of oropharyngeal lesions, the query remains if a screening method exists that can detect early-stage malignant disease. In a study conducted by Fakhry et al., the authors attempted to find a “Papanicolaou test equivalent” which used cytology brushes to collect cell samples that would be useful for screening and detection of HPV-associated oropharyngeal carcinoma. However, they concluded that in the absence of clinically visible lesions, cytologic evaluation as a screening method is not possible [6].

Anogenital cancers of the cervix, anus, vagina, vulva, and penis have a multi-step progression from pre-cancerous lesions to cancerous lesions. Subsequently, this allows for the success of a secondary screening modality. There is no HPV-induced precancerous lesion that has been identified. The presence of visible oral lesions and a known HPV oral infection remains as future indicators of cancer risk. However, only a few studies have thus estimated the percentage of HPV infections that persist and progress to oral cancer. While the incidence of oral cancer is low among low-risk healthy individuals (<3%) and higher in high-risk (HIV) populations (20%), the absolute risk of cancer in these individuals is unknown [7]. By analogy to cervical cancers in which persistent HPV infection heightens cancer risk, this has not yet been conferred for oropharyngeal cancer.

Oropharyngeal cancers are thought to initiate in the deep crypts of the tonsillar bed. As a result, accessing these deep crypts may not be possible for cytologic evaluation and a superficial brush biopsy is rendered insufficient for sampling the necessary cells. This is analogous to the limitation of cervical pap smear for screening cervical adenocarcinoma, which arises from the glandular cells of the endocervix.

Despite its growing burden, difficulties exist in secondary prevention of oropharyngeal cancers. This brings to the forefront the importance of primary prevention through prophylactic HPV vaccination and abstinence from smoking and alcohol. The mechanism involved in vaccine effectiveness in the anogenital tract is likely the same for the oropharyngeal tract. However, due to the inability to screen for premalignant lesions in the oral cavity, the relative rarity of oropharyngeal cancer, and the prolonged latent period between HPV infection and clinical cancer development, proving the effectiveness of such vaccines in the prevention of oral cancer will be challenging. Current-generation HPV vaccines (HPV 16/18) have the potential to prevent >90% of HPV-positive oropharyngeal cancers. Despite the safety and potential benefit of these vaccines, in the United States, uptake has proven to be poor, primarily due to low physician recommendation and low public awareness. According to the Centers for Disease Control, less than half of the teens in the United States were vaccinated with the HPV vaccine in 2017 [8]. Strategies must be implemented to increase HPV vaccination rates. The disparities in HPV vaccination may be curbed by providing public health education and encouraging physicians to be more proactive during medical visits and promote vaccination.

## Conclusions

Until an effective modality is available for early detection of oral cancer, innovative approaches to improve HPV vaccination status is crucial. Since HPV-related oral cancer has a good response to treatment if found early, prevention at an early stage is of paramount importance. Prevention strategies aimed at alcohol and tobacco cessation are also important since these remain the major risk factors for the occurrence of head and neck cancers across the world.
